# Assessment of the Impact of Bone Quality and Abutment Configuration on the Fatigue Performance of Dental Implant Systems Using Finite Element Analysis (FEA)

**DOI:** 10.3390/jpm14101040

**Published:** 2024-09-28

**Authors:** Meryem Erdoğdu, Mehmet Gökberkkaan Demirel, Reza Mohammadi, Neslihan Güntekin

**Affiliations:** 1 Department of Prosthodontics Dental Therapy, Faculty of Dentistry, Necmettin Erbakan University, 42090 Konya, Turkey; kaandemirel@erbakan.edu.tr (M.G.D.); nguntekin@erbakan.edu.tr (N.G.); 2 Faculty of Dentistry, Necmettin Erbakan University, 42090 Konya, Turkey; reza.mohammadi@ogr.erbakan.edu.tr

**Keywords:** bone quality, dental implants, dental software, fatigue performance, finite element analysis

## Abstract

Background and Objectives: The aim of this study was to evaluate the influence of abutment angulation, types, and bone quality on fatigue performance in dental implant systems. Materials and Methods: Three-dimensional models of maxillary 3-unit fixed implant-supported prostheses were analyzed. Abutments with different angles and types were used. Healthy bone (Hb) and resorbed bone (Rb) were used. Conducted on implants, a force of 150 N was applied obliquely, directed from the palatal to the buccal aspect, at a specific angle of 30 degrees. The stress distribution and fatigue performance were then evaluated considering the types of bone used and the angles of the three different abutments. The simulation aspect of the research was carried out utilizing Abaqus 2020 software. Results: In all models, fatigue strengths in healthy bone were higher than in resorbed bone. Maximum stress levels were seen in models with angled implants. In almost all models with resorbed bone, fatigue performances were slightly lower. Conclusions: Increasing the abutment angle has been shown to increase stress levels and decrease fatigue performance in the adjacent bone and along the implant–abutment interface. In general, implants applied to healthy bone were found to have a higher success rate. It has also been suggested that multiunit abutments have beneficial effects on stress distribution and fatigue performance compared to resin cemented abutments. The type or angle of abutment and the quality of the bone can lead to biomechanical changes that affect the force distribution within the bone structure surrounding the implant. Clinicians can influence the biomechanical environment of the implant site by varying the abutment angle and type to suit the condition of bone health, potentially affecting the long-term success of implant treatment.

## 1. Introduction

Implant angulation significantly influences the success of implant treatments by affecting impression accuracy, stress distribution, and the stability of implants, with studies indicating that optimal angulation enhances prosthetic outcomes and reduces strains during impression removal. Furthermore, the choice of impression techniques and materials, as well as the use of intraoral scanners, also play critical roles in determining the accuracy of implant casts. In the presence of angled implants, the evaluation of straight/angled multiunit and cemented abutments reveals significant differences in fatigue performance and stress distribution, which are critical for the long-term success of implant-supported restorations. Angled multiunit abutments are particularly advantageous as they can effectively manage the stress distribution resulting from the angulation of the implants. Research indicates that these abutments can help align the prosthetic components more favorably with the occlusal forces, thereby reducing stress concentrations at the implant-bone interface [1]. Finite element analysis has shown that angled multiunit abutments can lead to improved stress distribution patterns, which may mitigate the risk of bone resorption and enhance the overall stability of the implant [2]. Conversely, cemented abutments, while providing excellent occlusal integrity and aesthetics due to the absence of a screw access hole, may present challenges in terms of stress distribution. The cement layer can facilitate a passive fit, which is beneficial for distributing occlusal forces; however, the reliance on cement also introduces the risk of residual cement leading to peri-implant diseases [3]. The angle of the abutment can significantly influence the stress distribution, with higher angles potentially leading to increased stress on the surrounding bone, which could compromise the longevity of the restoration [4]. In summary, while angled multiunit abutments may offer superior stress management and fatigue performance in the context of angled implants, cemented abutments provide aesthetic advantages but may be more susceptible to complications related to stress distribution and residual cement. The choice between these abutment types should be guided by a thorough assessment of the clinical scenario, including the specific angulation of the implants and the desired functional and aesthetic outcomes.

The placement of dental implants in healthy bone versus resorbed bone has significant implications for fatigue performance and stress distribution, which are crucial for the long-term success of implant-supported restorations. Implants placed in healthy bone typically exhibit superior mechanical properties due to the higher density and quality of the surrounding bone, which allows for better load distribution and reduced stress concentrations at the implant-bone interface. Studies indicate that the elastic modulus of cortical bone contributes to stress concentration at the crestal level, which can enhance the stability and longevity of the implant [5]. This is particularly important as high stress and strain values at the crestal bone level can lead to complications such as bone resorption and implant failure if not managed properly [6]. In contrast, implants placed in resorbed bone often face challenges related to reduced bone quality and density, which can lead to increased stress concentrations and fatigue failure. Research has shown that the mechanical properties of resorbed bone differ significantly from healthy bone, resulting in less effective load distribution and higher susceptibility to stress shielding [7]. For instance, finite element analyses have demonstrated that vertical forces in resorbed bone can lead to uniform stress distribution along the implant–bone interface, while oblique forces may cause shear forces and bending moments, concentrating stress at the implant neck and surrounding bone [8]. This can exacerbate the risk of marginal bone loss and implant failure, particularly in cases where the bone has been augmented or grafted [9]. Furthermore, the choice of graft material and surgical technique can influence the outcomes of implants in resorbed bone. Autogenous bone grafts have been shown to provide better integration and stability compared to allografts or xenografts, leading to improved stress distribution and fatigue performance [10]. Additionally, the use of guided bone regeneration techniques can help mitigate the risks associated with resorbed bone by promoting the formation of new bone around the implant, thereby enhancing its mechanical stability [11].

While the literature provides insight into the advantages and considerations of angled abutment use in cases of angulated implant and resorbed bone, there is a gap in understanding the specific effects of the types of angled abutments that can be used in the presence of resorbed bone. Further research focusing on the interaction between abutment design, implant angle, and Rb is needed to improve clinical decision-making in implant dentistry. Based on various findings from previous studies on the effect of different abutment angulations and types on fatigue performance of implant components on Hb or Rb, it has been concluded that more research is needed in this area. The primary objective of this study was to evaluate the effect of various abutment angulations and types on the stress distribution and fatigue performance on the implant and adjacent tissues using FEA for implant-supported prostheses on Hb or Rb. The null hypothesis of the study is that the presence of Rb will not affect the fatigue performance of the implant component and the abutment angulation and type will also not affect fatigue performance.

## 2. Materials and Methods

### 2.1. Preparation of Specimens

**Figure 1:**
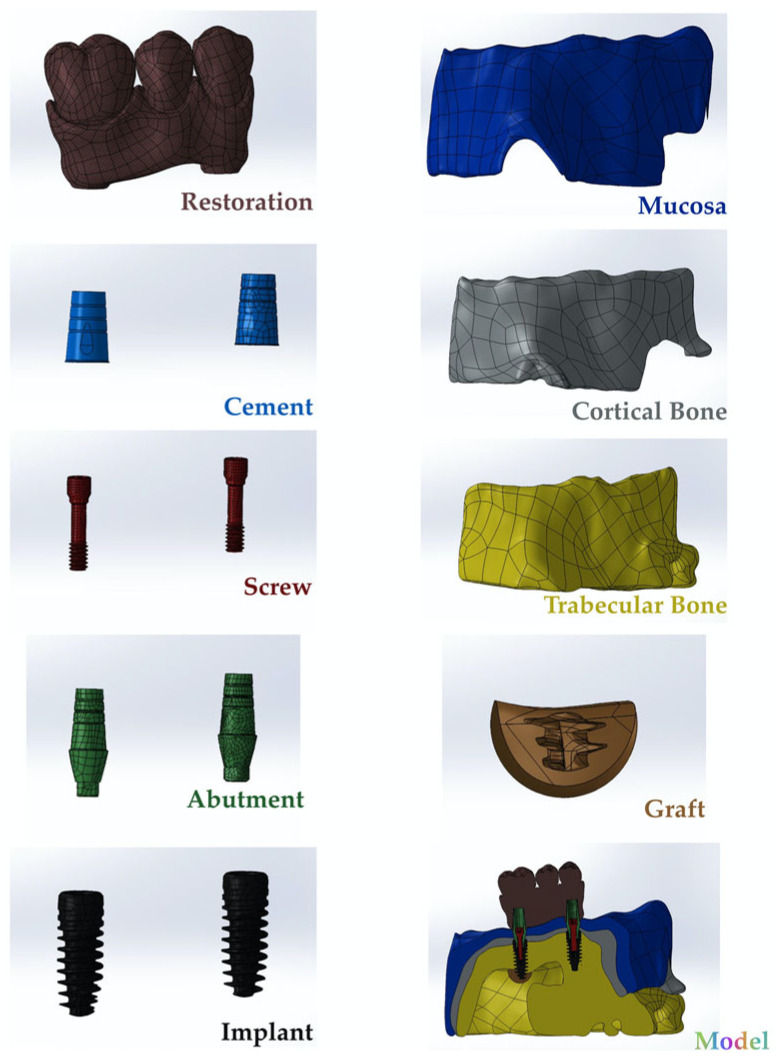
The finalized versions of the generated models.

**Figure 2:**
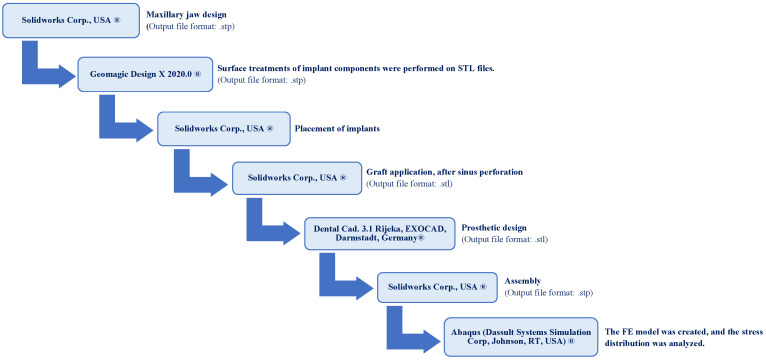
Schematic workflow of the methodology used.

In this study, multiunit and resin-cemented abutment types of Bil implant company (İstanbul, Türkiye) were used. Monolithic zirconia was used as restoration material. Straight (3LSRAGH2NP, new ⌀3.7 mm implant L 10 mm), 15° (abutment screw NP, BLSR156H2ANP, new ⌀3.7 mm implant L 10 mm), and 30° (abutment screw NP, BLSR306H2ANP, new ⌀3.7 mm implant L 10 mm) abutments of the company were used for the multiunit design. Additionally, for the resin-cemented type, straight (abutment screw NP, new ⌀3.7 mm implant L 10 mm, PAD35GH2AH55NP), 15° (abutment screw NP, new ⌀3.7 mm implant L 10 mm, PAA156H2AH7NP), and 25° (abutment screw NP, new ⌀3.7 mm implant L 10 mm, PAA25GH2AH7NP) abutments were used. The groups used in the analysis were as follows: multiunit 0 degree (Mu0), multiunit 15 degree (Mu15), multiunit 30 degree (Mu30), cemented 0 degree (C0), cemented 15 degree (C15), and cemented 25 degree (C25). To prepare the specimens, a model was created to simulate the maxillary jawbone (trabecular and cortical bone and mucosa) of an edentulous patient using the assembly module of the Solidworks 2013 program (Solidworks Corp., Waltham, MA, USA). For this study, 3 tooth spaces were considered. The 1st and 2nd premolars and the 1st molar were considered as missing tooth regions. One implant was placed in the 1st premolar area and another implant was placed in the 1st molar area. The 2nd premolar was left empty and a 3-unit bridge supported by 2 implants was planned. The implants were positioned in the generated STP file based on information provided by Bil Implant Company. In the insert section, the indent command in the feature section was used to create the cavity of the implant in the bone. At this stage, during the implant placement, sinus perforation occurred in implant number 6. In the area where the implant perforated the sinus, the graft material given in Table 1 was used to completely cover the perforation area. Following the creation of STL files, the prosthesis designs were developed using Exocad (Dental Cad. 3.1 Rijeka, EXOCAD, Darmstadt, Germany). These STL files were then imported into Geomagic Design X (Geomagic Design X 2020.0), where adjustments were made to produce new STP files. The prostheses were subsequently positioned on the implants in the model. For the resin-cemented restorations, a 40-micrometer cement gap was filled with resin cement. The finalized models were saved as STP files and imported into Abaqus (2020 Dassault Systems Simulation Corp., Johnston, RI, USA) for finite element analysis (FEA) (Figure 1). In the Abaqus program, the material properties were first entered in the property section. The analysis type was specified as static, general, and direct cyclic in the step section, with the number of cycles set to 1000 in the direct cyclic step. In the interaction section, all contacts, except for the one between the screw and the implant, were defined as tie constraints (to simulating osseointegration between the framework and porcelain, composite and prosthesis, bone and mucosa, and implant and bone). The connections between the screw parts were modeled with torque properties. Loading and boundary conditions were then applied in the load section. Finally, the mesh was created in the mesh section of the Abaqus program. In order to improve the accuracy of the group results, mesh adjustment was made throughout the run and repetitions were performed for each group in order to obtain the final accurate results (Figure 2). Material properties for all components included in the study were defined (Table 1). The chosen properties reflect the typical mechanical behavior of every material as documented in previous studies, as also mentioned in Table 1.

### 2.2. Prediction of Stress Distribution

In this experimental setup, a force of 150 N was exerted on the occlusal table, which was inclined from the palatal to the buccal direction at an angle of 30 degrees. The maxillary bone was considered encastre, indicating that it was constrained from any movement or rotation in all planes. The study assumed full osseointegration of the implants with the bone, ensuring no motion of the implant components on the implant surface. To assess the quality of integration, a frictional contact model was employed between the abutment, fixture, abutment screw interface, and fixture, with a friction coefficient of 0.3 being specified for the interactions.

**Figure 3:**
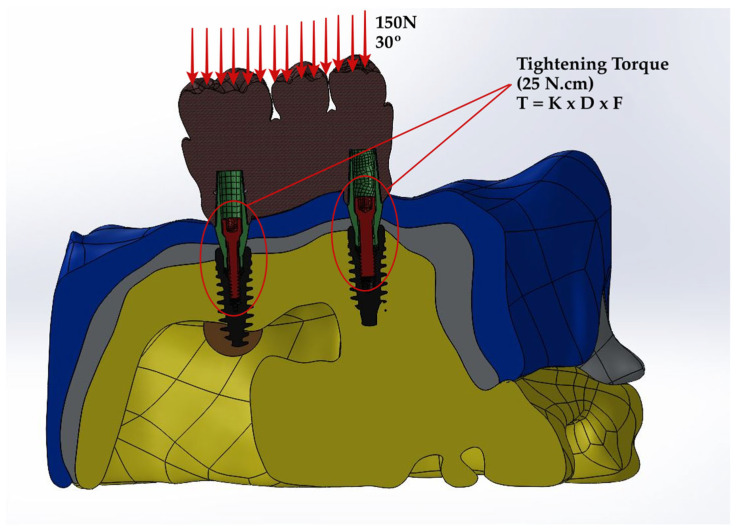
Screw preloading with the bone block fixed along the axes.

The formula for the torque applied on the screw is presented below;

(1) T=K×D×F,

*T* represents the screw torque moment (N·cm), *D* the screw diameter (m), *F* the screw preload (N), and *K* the screw factor or torque coefficient (commonly with a value of 0.2). By utilizing the specified equation, the preload value of the screw can be accurately computed based on the applied tightening moment. In this study, the screw preload value was calculated as 781 N by using a tightening moment of 25 N·cm (Figure 3). Subsequently, stress values were derived from this process, specifically in the form of von Mises stress values (vMSs).

### 2.3. Prediction of Fatigue Performance

To ensure the enduring success of implants over the long term, it is imperative to attain the highest level of fatigue performance. To evaluate this critical aspect, either physical testing or fatigue analysis can be employed as assessment methods. In the present research endeavor, a series of equations were utilized within finite element (FE) modeling to assess and predict the fatigue performance of the implants.

(2) $$\frac{\gamma }{2}+\frac{{∆\varepsilon }_{N}}{2}=1.65\frac{\sigma_{f}^{\prime }-\sigma_{N,m}}{E}{\left(2N_{f}\right)}^{b}+1.75\varepsilon_{f}^{\prime }{\left(2N_{f}\right)}^{c}.$$

*σ_N_*_,*m*_ corresponds to the normal stress on the critical plane, 2*N_f_* to the number of reversals to crack initiation, γ/2 to the shear strain amplitude, Δ*ε_N_* to the normal strain on the critical plane, $\sigma_{f}^{\prime }$ to the fatigue strength coefficient, $\varepsilon_{f}^{\prime }$ to the normal fatigue ductility coefficient, *E* to the elastic modulus, *c* to the fatigue ductility exponent, and *b* to the fatigue strength exponent. The fatigue ductility exponent and coefficient are derived from the Coffin–Manson law [18].

(3) $$\varepsilon_{P}=\varepsilon_{f}^{\prime }{\left(2N_{f}\right)}^{c}.$$

$\varepsilon_{P}$ represents plastic strain.

The fatigue strength exponent and coefficient come from Basquin’s law:

(4) $$\varepsilon_{e}=\frac{\sigma_{a}}{E}=\frac{\sigma_{f}^{\prime }}{E}{\left(2N_{f}\right)}^{b}.$$

(5) $$\sigma_{a}=\frac{∆\sigma }{2}=\frac{\left(\sigma_{max}-\sigma_{min}\right)}{2}.$$

$\varepsilon_{e}$ is equivalent to the elastic component of the cyclic strain amplitude; and $\sigma_{a}$ is equivalent to the cyclic stress amplitude.

The material characteristics were estimated utilizing the Seeger method, which involves adjusting the conventional monotonic ultimate tensile stress (UTS) [19]. The values corresponding to titanium’s relative parameters based on the Seeger method are presented in Table 2 for reference.

## 3. Results

### 3.1. Stress Distribution

#### 3.1.1. Stress Values on the Implants

When the vMSs obtained as a result of the study were analyzed, the highest vMS on the implant in the Hb premolar region was seen in C25, while the lowest vMS was seen in Mu0. VMSs increased in direct proportion to the increase in angle in the groups. Multiunit abutments obtained lower vMS than cemented abutments at all angles.

When the vMS on the implants in the molar region on Hb was analyzed, the lowest vMS was found in Mu0, while the highest vMS was found in C25, as it was also in the premolar region. Again, in the molar region, an increase in vMS was also detected with increasing angle. Multiunit abutments obtained lower vMS than cemented abutments.

When both Hb regions were analyzed, vMS in the molar zone was found to be higher in each abutment type.

**Figure 4:**
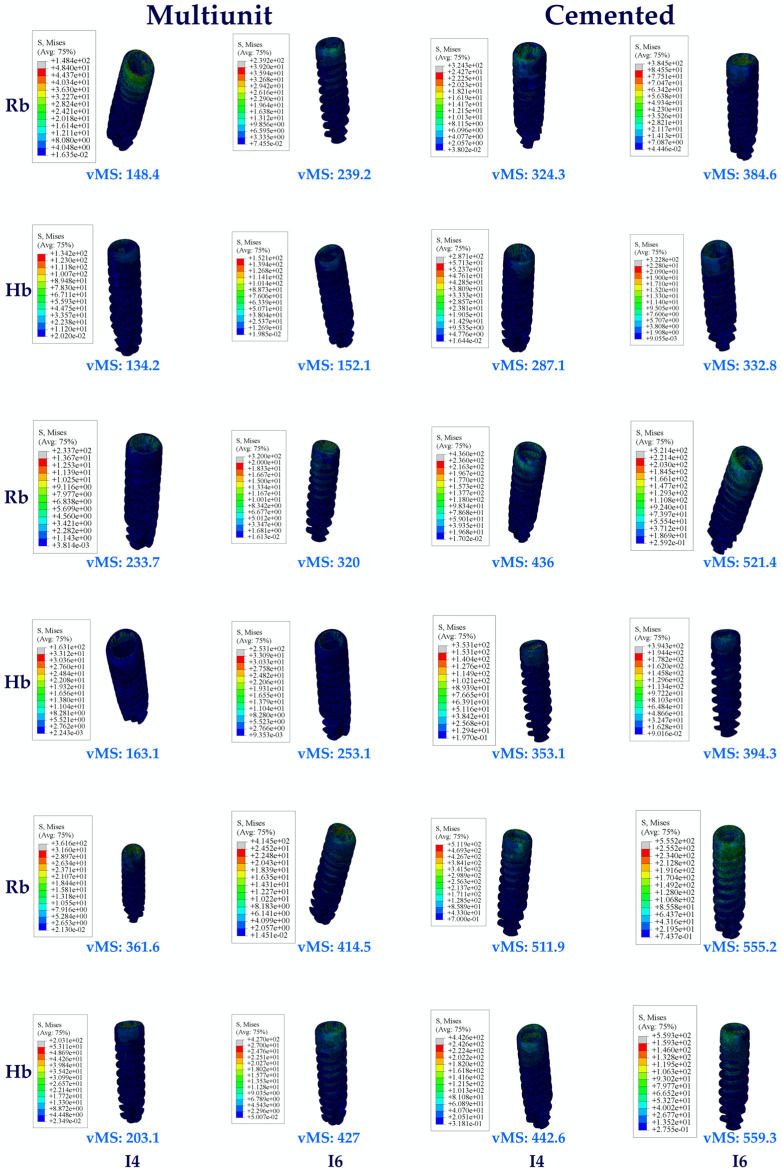
Von Mises stress on the implant. vMS: von Mises stress; I4: implant in the premolar area; I6: implant in the molar area; Hb: healthy bone; Rb: resorbed bone.

The same vMS distributions were obtained in Rb models as in Hb models. Numerically higher vMSs were obtained in the Rb models compared to the Hb models (Figure 4).

#### 3.1.2. Stress Values on the Abutments

Regarding the vMS on the abutments in the Hb model, the highest vMS on the abutment in the premolar region was seen in C25, while the lowest vMS was seen in Mu0. An increase in stress values was observed in direct proportion to the increase in angle in the groups. Multiunit abutments obtained lower vMS than cemented abutments at all angles.

When the vMS on the abutments in the molar region on Hb was analyzed, it was found that the lowest vMS was in Mu0, while the highest vMS was in C25, as in the premolar region. Again, in the molar region, an increase in vMS was also detected with increasing angle. Multiunit abutments obtained lower vMS than cemented abutments.

When both Hb regions were analyzed, vMS in the molar zone was higher in each abutment type.

**Figure 5:**
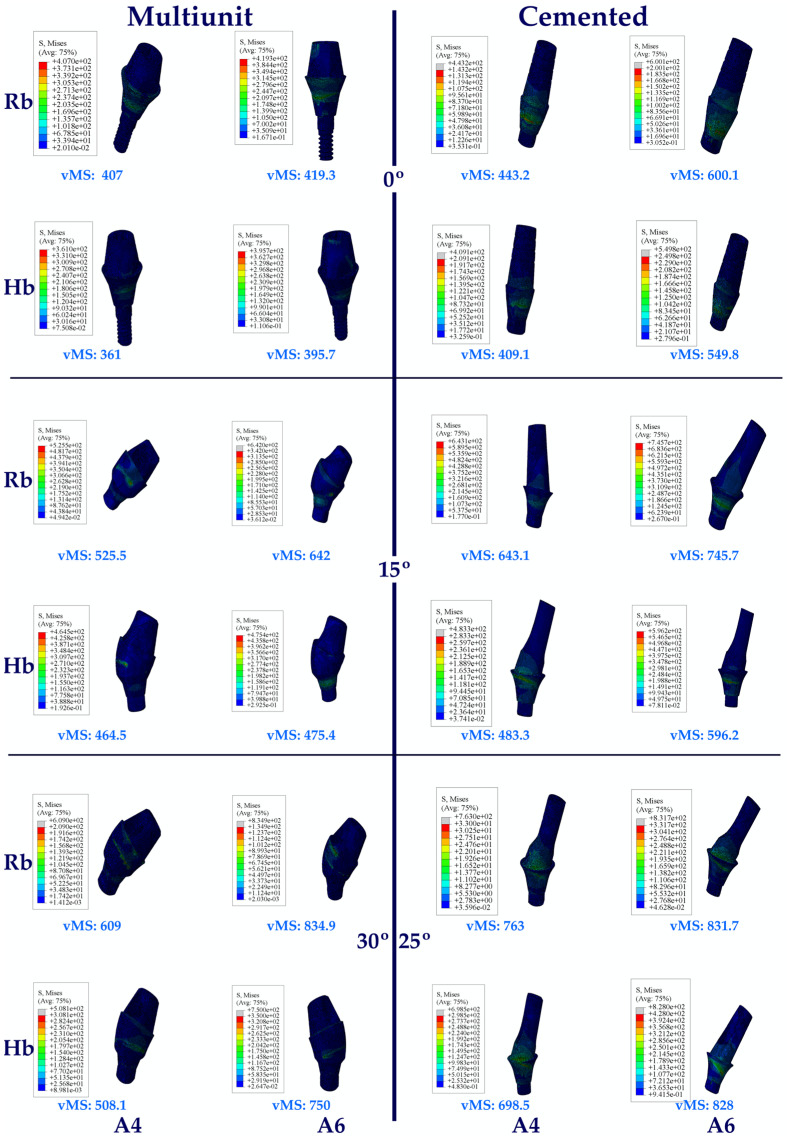
Von Mises stress on the abutment. vMS: von Mises stress; A4: abutment in the premolar area; A6: abutment in the molar area; Hb: healthy bone; Rb: resorbed bone.

The same vMS distributions were obtained in Rb models as in Hb models. Numerically higher vMSs were obtained in the Rb models compared to the Hb models (Figure 5).

#### 3.1.3. Stress Values on the Abutment Screw

The highest vMS in abutment screws in the Hb models was found at C25. In addition, the lowest vMS was observed at C0. An increase in vMS values was also observed with increasing angle.

When the vMS of the abutment screws in the molar region on Hb was analyzed, similar results were found with the premolar region. The highest vMS was found at C25. In addition, the lowest vMS was observed at C0. An increase in vMS values was also observed with increasing angle.

When both Hb regions were examined, it was found that the abutment screw vMSs in the molar region were higher.

**Figure 6:**
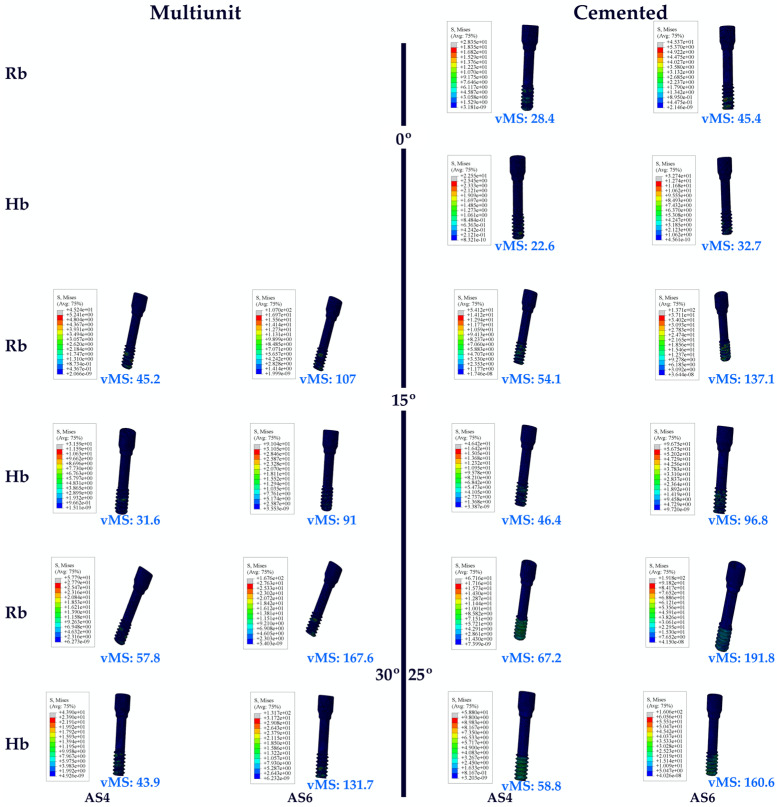
Von Mises stress on the abutment screw. vMS: von Mises stress; AS4: abutment screw in the premolar area; AS6: abutment screw in the molar area; Hb: healthy bone; Rb: resorbed bone.

The same vMS distributions were obtained in Rb models as in Hb models. Numerically higher vMSs were obtained in the Rb models compared to the Hb models (Figure 6).

#### 3.1.4. Stress Values on the Occlusal Screw

When the occlusal screw VMSs in Hb multiunit restorations were analyzed, the lowest values were observed at Mu0 and the highest at Mu30 in molar and premolar regions. An increase in vMSs was also observed with increasing angle. VMSs in the molar region obtained lower values compared to premolar regions.

**Figure 7:**
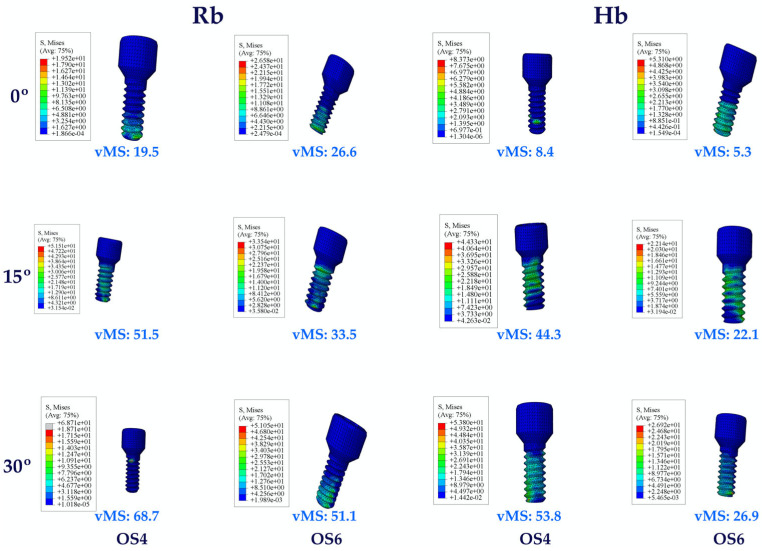
Von Mises stress on the occlusal screw. vMS: von Mises Stress; OS4: occlusal screw in the premolar area; OS6: occlusal screw in the molar area; Hb: healthy bone; Rb: resorbed bone.

The same vMS distributions were obtained in Rb models as in Hb models. Numerically higher vMSs were obtained in the Rb models compared to the Hb models (Figure 7).

#### 3.1.5. Stress Values on the RESIN Cement

When the vMSs on the resin cement in the Hb premolar region were analyzed, the highest vMS was observed at C0, while the lowest vMS was detected at C25. In inverse proportion to the increase in angle, a decrease in vMS was observed.

When analyzing the resin cement vMSs in the Hb molar region, the opposite results were obtained compared to the premolar region. The highest vMS was observed at C25 and the lowest at C0. An increase in vMS was also observed with increasing angle.

When both Hb regions were analyzed together, higher vMSs were obtained in the molar region.

**Figure 8:**
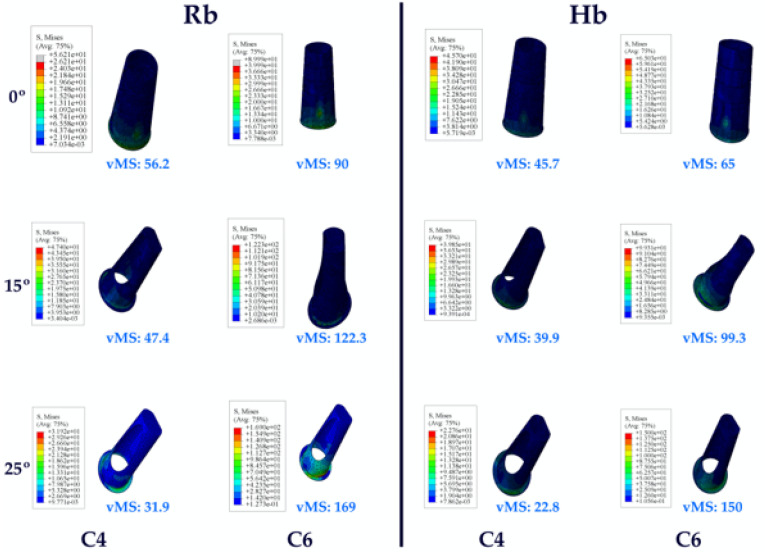
Von Mises stress on the cement. vMS: von Mises stress; C4: cement in the premolar area; C6: cement in the molar area; Hb: healthy bone; Rb: resorbed bone.

The same vMS distributions were obtained in Rb models as in Hb models. Numerically higher vMSs were obtained in the Rb models compared to the Hb models (Figure 8).

#### 3.1.6. Stress Values on the Prosthetic Restoration

When the prosthetic restoration vMSs in the Hb groups were analyzed, the highest value was seen in Mu0. The lowest value was detected at C25. Increasing the angle resulted in a decrease in vMSs.

**Figure 9:**
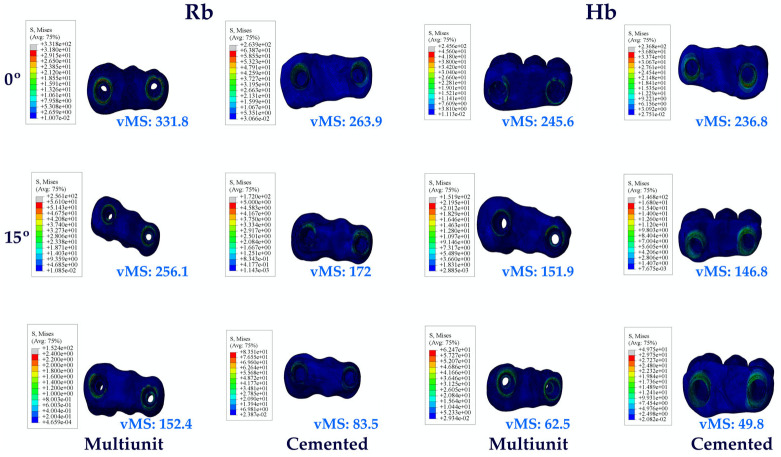
Von Mises stress on the prosthetic restoration. vMS: von Mises Stress; Hb: healthy bone; Rb: resorbed bone.

The same vMS distributions were obtained in Rb models as in Hb models. Numerically higher vMSs were obtained in the Rb models compared to the Hb models (Figure 9).

#### 3.1.7. Stress Values on the Cortical Bone and Bone Graft

Looking at the vMSs on Hb, the highest vMS was observed at C25, while the lowest vMS was observed at Mu0. Again, an increase in vMSs was observed with increasing angle.

The same vMS distributions were obtained in Rb models as in Hb models. Numerically higher vMSs were obtained in the Rb models compared to the Hb models.

**Figure 10:**
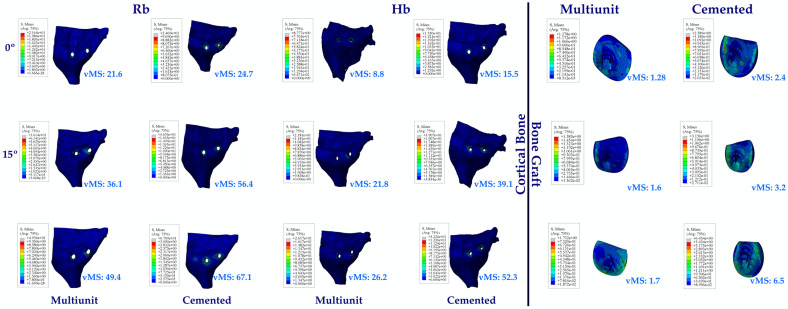
Von Mises stress on cortical bone and bone graft. vMS: von Mises stress; Hb: healthy bone; Rb: resorbed bone.

Since no perforation was observed during implant placement in the premolar region, no bone graft was used in this region. Perforation occurred in the molar area during implant placement. When the bone graft vMS in the molar region was analyzed, the highest vMS was observed in C25, while the lowest vMS was observed in Mu0. An increase in vMS was also observed with increasing angle. While lower vMSs were observed in Mu abutments, higher vMSs were observed in C abutments (Figure 10).

### 3.2. Fatigue Performances

#### 3.2.1. Fatigue Performance of Implant

**Figure 11:**
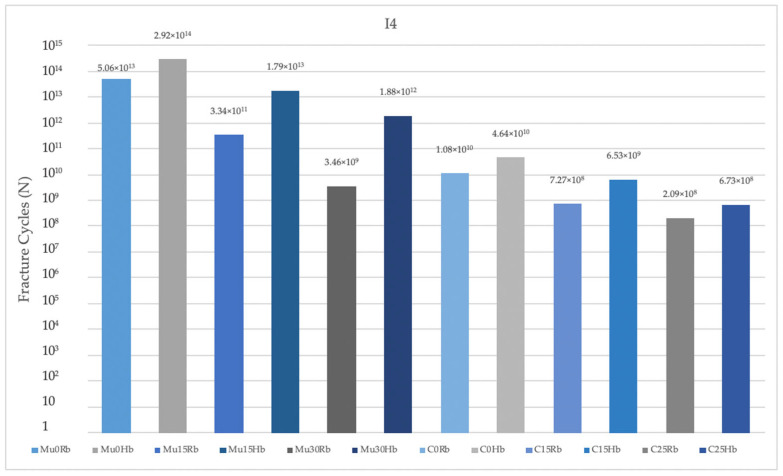
Fracture cycles of implants in the premolar region. I4: implant in the premolar area; Mu0Rb: multiunit 0 degree resorbed bone; Mu0Hb: multiunit 0 degree healthy bone; Mu15Rb: multiunit 15 degree resorbed bone; Mu15Hb: multiunit 15 degree healthy bone; Mu30Rb: multiunit 30 degree resorbed bone; Mu30Hb: multiunit 30 degree healthy bone; C0Rb: cemented 0 degree resorbed bone; C0Hb: cemented 0 degree healthy bone; C15Rb: cemented 15 degree resorbed bone; C15Hb: cemented 15 degree healthy bone; C25Rb: cemented 25 degree resorbed bone; C25Hb: cemented 25 degree healthy bone.

As a result of this study, fatigue performances obtained in the Hb model were analyzed. The highest fatigue performance was observed in Mu0 for implants in the premolar region. The lowest value was observed in C25. A decrease in fatigue performance was observed inversely proportional to the increase in angles. The highest fatigue performances were observed in implants with Mu abutments (Figure 11).

**Figure 12:**
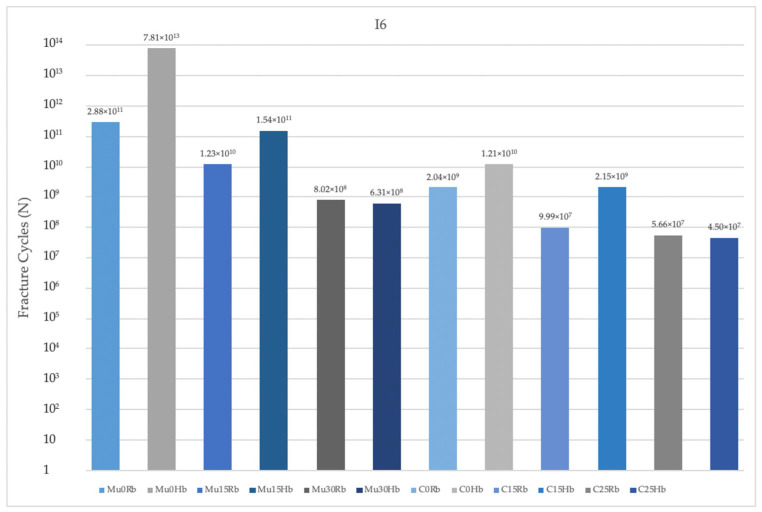
Fracture cycles of implants in the molar region. I6: implant in the molar area; Mu0Rb: multiunit 0 degree resorbed bone; Mu0Hb: multiunit 0 degree healthy bone; Mu15Rb: multiunit 15 degree resorbed bone; Mu15Hb: multiunit 15 degree healthy bone; Mu30Rb: multiunit 30 degree resorbed bone; Mu30Hb: multiunit 30 degree healthy bone; C0Rb: cemented 0 degree resorbed bone; C0Hb: cemented 0 degree healthy bone; C15Rb: cemented 15 degree resorbed bone; C15Hb: cemented 15 degree healthy bone; C25Rb: cemented 25 degree resorbed bone; C25Hb: cemented 25 degree healthy bone.

In the molar region, the highest value was seen in Mu0. The lowest value was seen in C25. Increasing the angle caused a decrease in fatigue performance values. The highest fatigue performances were obtained in implants with Mu abutments (Figure 12).

When both regions were analyzed, lower fatigue performance values were obtained in the molar region.

When the fatigue performance of the implants in the Rb models was analyzed, the highest values were seen in Mu0 in both molar and premolar regions and the lowest values were seen in C25 also in both molar and premolar regions, as in the Hb model. Again, in the Rb region, fatigue performance decreased with increasing angle. The highest performance values were obtained in implants with Mu abutments.

#### 3.2.2. Fatigue Performance of Abutment

**Figure 13:**
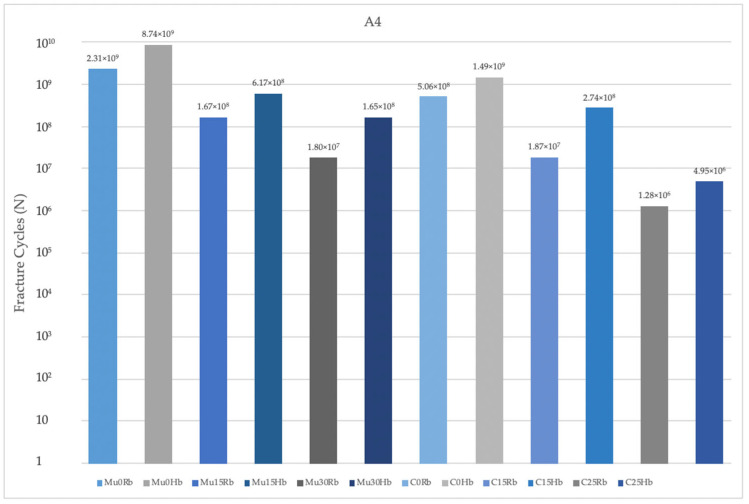
Fracture cycles of abutments in the premolar region. A4: abutment in the premolar area; Mu0Rb: multiunit 0 degree resorbed bone; Mu0Hb: multiunit 0 degree healthy bone; Mu15Rb: multiunit 15 degree resorbed bone; Mu15Hb: multiunit 15 degree healthy bone; Mu30Rb: multiunit 30 degree resorbed bone; Mu30Hb: multiunit 30 degree healthy bone; C0Rb: cemented 0 degree resorbed bone; C0Hb: cemented 0 degree healthy bone; C15Rb: cemented 15 degree resorbed bone; C15Hb: cemented 15 degree healthy bone; C25Rb: cemented 25 degree resorbed bone; C25Hb: cemented 25 degree healthy bone.

When the fatigue performances of the abutments used in the premolar region in the Hb models were examined, the highest values were found in Mu0. The lowest values were seen in C25. Increasing the angle caused a decrease in fatigue performances. The highest fatigue performances were observed in models using Mu abutments (Figure 13).

**Figure 14:**
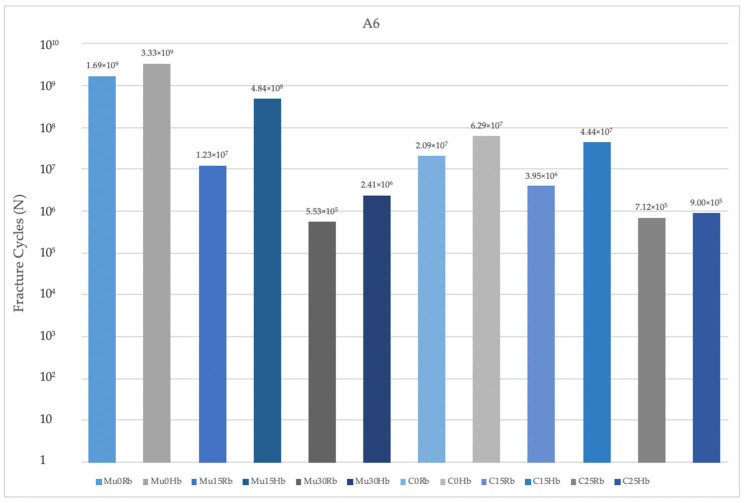
Fracture cycles of abutment in the molar region. A6: abutment in the molar area; Mu0Rb: multiunit 0 degree resorbed bone; Mu0Hb: multiunit 0 degree healthy bone; Mu15Rb: multiunit 15 degree resorbed bone; Mu15Hb: multiunit 15 degree healthy bone; Mu30Rb: multiunit 30 degree resorbed bone; Mu30Hb: multiunit 30 degree healthy bone; C0Rb: cemented 0 degree resorbed bone; C0Hb: cemented 0 degree healthy bone; C15Rb: cemented 15 degree resorbed bone; C15Hb: cemented 15 degree healthy bone; C25Rb: cemented 25 degree resorbed bone; C25Hb: cemented 25 degree healthy bone.

Fatigue values in the molar region also matched those in the premolar region. When the two regions were analyzed, lower fatigue performances were obtained in the molar region (Figure 14).

Abutment fatigue performances in Rb models were also analyzed. The highest values were observed at Mu0 in both regions. The lowest values were seen at C25 in the premolar region and Mu30 in the molar region. In the Rb models, the fatigue performance decreased with increasing angle.

#### 3.2.3. Fatigue Performance of Abutment Screw

**Figure 15:**
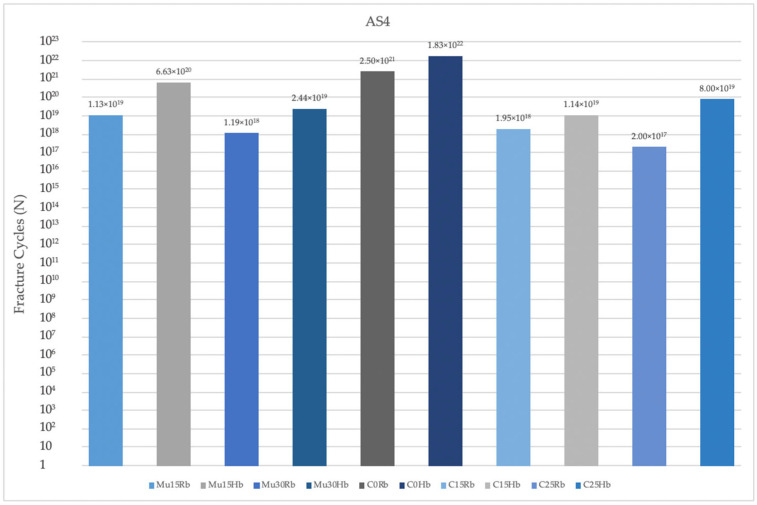
Fracture cycles of abutment screw in the premolar region. AS4: abutment screw in the premolar area; Mu15Rb: multiunit 15 degree resorbed bone; Mu15Hb: multiunit 15 degree healthy bone; Mu30Rb: multiunit 30 degree resorbed bone; Mu30Hb: multiunit 30 degree healthy bone; C0Rb: cemented 0 degree resorbed bone; C0Hb: cemented 0 degree healthy bone; C15Rb: cemented 15 degree resorbed bone; C15Hb: cemented 15 degree healthy bone; C25Rb: cemented 25 degree resorbed bone; C25Hb: cemented 25 degree healthy bone.

The fatigue performances of abutment screws used in Hb models were analyzed. The highest fatigue performances were recorded at C0 in both regions. The lowest fatigue performance value in the premolar region was seen in C15, while in the molar region it was seen in C25. In both regions, the increase in angle caused a decrease in fatigue performance values. Lower fatigue performances were obtained in the molar region than in the premolar region (Figure 15).

**Figure 16:**
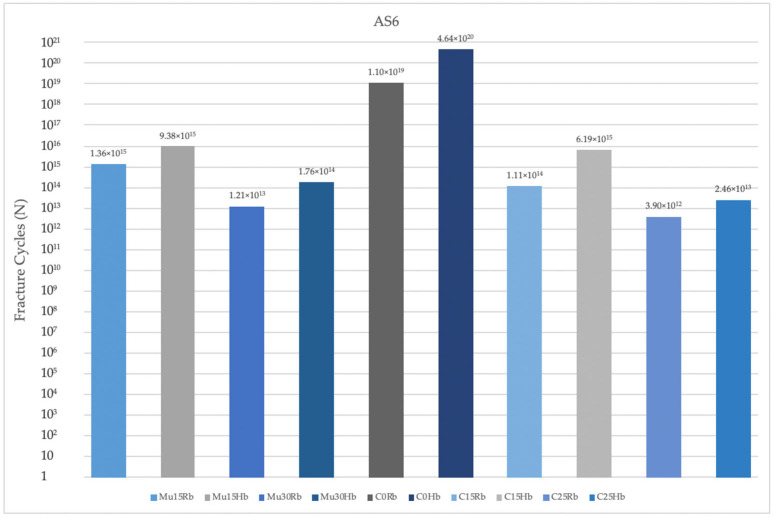
Fracture cycles of abutment screw in the molar region. AS6: abutment screw in the molar area; Mu15Rb: multiunit 15 degree resorbed bone; Mu15Hb: multiunit 15 degree healthy bone; Mu30Rb: multiunit 30 degree resorbed bone; Mu30Hb: multiunit 30 degree healthy bone; C0Rb: cemented 0 degree resorbed bone; C0Hb: cemented 0 degree healthy bone; C15Rb: cemented 15 degree resorbed bone; C15Hb: cemented 15 degree healthy bone; C25Rb: cemented 25 degree resorbed bone; C25Hb: cemented 25 degree healthy bone.

When the abutment screws were also examined in Rb models, in the molar region, the highest fatigue performances were seen at C0, while the lowest values were seen at C25 in both regions. In both regions, the increase in angle caused a decrease in fatigue performance values. Lower fatigue performances were obtained in the molar region than in the premolar region (Figure 16).

#### 3.2.4. Fatigue Performance of Occlusal Screw

**Figure 17:**
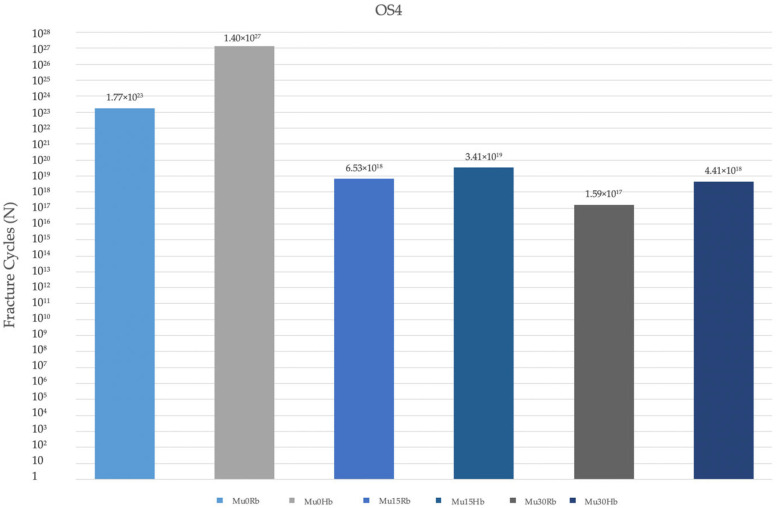
Fracture cycles of occlusal screw in the premolar region. OS4: occlusal screw in the premolar area; Mu0Rb: multiunit 0 degree resorbed bone; Mu0Hb: multiunit 0 degree healthy bone; Mu15Rb: multiunit 15 degree resorbed bone; Mu15Hb: multiunit 15 degree healthy bone; Mu30Rb: multiunit 30 degree resorbed bone; Mu30Hb: multiunit 30 degree healthy bone.

**Figure 18:**
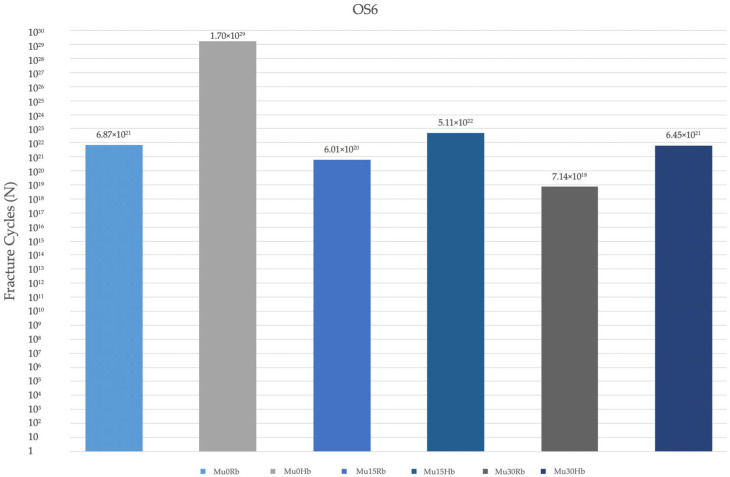
Fracture cycles of occlusal screw in the molar region. OS6: occlusal screw in the molar area; Mu0Rb: multiunit 0 degree resorbed bone; Mu0Hb: multiunit 0 degree healthy bone; Mu15Rb: multiunit 15 degree resorbed bone; Mu15Hb: multiunit 15 degree healthy bone; Mu30Rb: multiunit 30 degree resorbed bone; Mu30Hb: multiunit 30 degree healthy bone.

The fatigue performance of the occlusal screws used in the Hb and Rb models was analyzed. In both models, in both premolar and molar regions, the highest values were observed at Mu0, while the lowest values were observed at Mu30 (Figure 17). The fatigue performances decreased inversely proportional to the increase in angle. In general, fatigue performances in the molar region were lower in both models (Figure 18).

## 4. Discussion

This study investigated the impact of various abutment types and angulations on the stress distribution and fatigue performance of dental implant components, specifically focusing on the effects on Rb and Hb. The null hypothesis was rejected. The findings indicate that the presence of Rb significantly affects the stress distribution and fatigue resistance of the abutment–implant complex. Furthermore, the angulation of the implant also plays a critical role in modulating both stress distribution and fatigue performance. To investigate the effect of increasing angle on the fatigue performance of dental implants, it is essential to consider various factors that influence the fatigue strength of these implants. Proper implant design plays a crucial role in ensuring long-term fatigue performance [22]. Studies have shown that alterations in force application angles during fatigue testing can impact the number of load cycles before failure, highlighting the sensitivity of dental implants to changes in loading conditions [23]. Additionally, the length and loading angles of implants have been found to affect both static fracture and fatigue life, emphasizing the importance of these parameters in implant performance [24,25]. These studies clearly demonstrate the effects of implant loading angles and abutment design on fatigue life. The results of this study are in line with the literature. As a result of this study, an increase in stress values and a decrease in fatigue performance were observed because of an increase in angulation. Research by Arnold et al. [26] assessed the retentive characteristics of a new attachment system for hybrid dentures under various conditions, including implant angulations from 0° to 20°, emphasizing the importance of considering implant angulation in evaluating dental attachments. Teimoori et al. [27] also observed a notable decrease in retention for overdenture attachments at different angulations post cyclic loading, highlighting the impact of implant angulation on attachment stability. Moreover, implant angulation can affect the accuracy of implant impressions and the fit of implant-supported restorations. Studies by Alikhasi et al. [28] and Lopes et al. [29] demonstrated that increased implant angulation led to decreased impression accuracy, especially for highly non-axial implant angulations. Tan et al. [30] further reinforced the significance of considering angulation in prosthodontic procedures by investigating the effect of implant angulation on the positional accuracy of implant analogs in various models. Biomechanical aspects of implant angulation were explored by Ebadian et al. [31] and Brum et al. [32], who highlighted that implant angulations can reduce stresses on the implant and supporting bone when the load aligns with the angulation. Conversely, it was found that higher implant angulations resulted in increased stress peaks on the buccal aspect of the implant–tissue interface, underscoring the need to carefully consider angulation to prevent excessive stress concentrations. In addition, implants applied on Rb generally achieved higher vMS and decreased fatigue performance. These results are also similar to the literature. To assess the fatigue performance of implants applied on Hb versus Rb, it is crucial to consider the impact of bone quality on implant stability and longevity. Implants inserted in Rb may encounter challenges such as implant loosening due to bone resorption or fatigue failure from overload [33]. The stability of the implant–bone interface is vital for fostering osseointegration and preventing micromotion that could trigger bone resorption [34]. Moreover, the fatigue properties of implants are pivotal in their performance, with fatigue strength being a key determinant of the longevity of bone implants [35]. Research has indicated that various factors, including bone remodeling, stress distribution, and implant design, can influence the fatigue resistance of implants [36]. In the realm of bone augmentation, studies have shown that properties of the implant material, such as cement composition, can significantly affect the biomechanical performance and fatigue resistance of augmented bone [37]. Studies have shown that when implants are placed in areas with low crestal bone loss or resorbed bone, there is a reduction in compressive stress values at the cortical bone [38]. This reduction in stress distribution can lead to disuse atrophy, bone resorption, and implant loosening due to overload or fatigue failure of the bone [33,39]. Additionally, the resorption of the mineral phase around implants can decrease implant stability while increasing bone regeneration through mechanical stimulation [40]. Implant fatigue performance is a critical aspect in the success of dental implants, especially in cases where weak cortical bone is present. The cortical bone plays a significant role in the mechanical competence of implant anchorage [41]. When implants are subjected to oblique loads with high occlusal stress magnitudes, there is a risk of surpassing the elastic limit of the bone surrounding the implants, potentially leading to microfractures in the cortical bone [42]. Additionally, the placement of immediately loaded implants in cases with thin cortical bone and weak trabecular bone can induce extreme bone strains, increasing the risk of implant failure [43]. The stress distribution in the cortical bone and implant is influenced by various factors such as bone density and crestal cortical bone thickness. Studies have shown that bone with higher density is better able to distribute loads, while bone with lower density is more prone to implant failure due to overload [44]. Furthermore, low-density bone and thin crestal cortical bone at the implant placement site are identified as risk factors for overloads of immediate-loading implants [45]. Thinner cortical bone can cause larger micromotion and stress concentration, particularly when the cortical thickness is less than 0.3 mm [46]. Moreover, the controlled release of bone morphogenetic protein-2 from a microsphere coating applied to acid-etched titanium implants can increase biological bone growth in vivo, promoting cortical bone ingrowth with relatively high fixation strength [47]. The relationship between bone characteristics, such as cortical bone thickness and trabecular bone strength, and implant stability is crucial. Insufficient implant anchorage is a problem in mechanically weak or osteoporotic bone [48]. Studies have shown that the failure rate of implants is lower when placed in alveolar bone with thick cortical bone or dense spongy bone, compared to thin cortical bone and sparse spongy bone [49]. Additionally, the relative contribution of bone microarchitecture and matrix composition to implant fixation strength includes factors such as osteointegration volume/total volume, peri-implant trabecular bone volume fraction, and cortical thickness [50]. In the light of these studies, the higher vMS and lower fatigue performance values obtained in this study in the molar region can also be explained. When considering weak cortical bone in the molar versus premolar region and its impact on implant fatigue performance, it is crucial to understand the differences in cortical bone thickness and density at these sites. Studies have shown that cortical bone thickness varies significantly between different regions of the jawbone, with thinner cortical bone reported in the maxillary buccal and mandibular anterior regions, particularly around the canine-first premolar area [51]. Additionally, the cortical bone around dental implants plays a vital role in providing support against occlusal forces, with cortical bone contact improving the biomechanical performance of implants compared to trabecular bone support [52]. Furthermore, the biomechanical behavior of implants in the molar region has been studied extensively. Finite element analysis has demonstrated that stresses in nonsplinted and splinted restorations in the cortical bone of the angulated molar region are higher compared to straight molar implants, with splinted restorations leading to a better distribution of stresses in both the implant bodies and surrounding bone [31]. Subcrestal placement of implants has also been shown to decrease stress in the crestal cortical bone, regardless of apical anchorage, highlighting the importance of implant positioning for stress distribution [53]. The cortical bone thickness in the premolar region compared to the molar region has been a subject of interest in various studies. Park [54] noted that the lingual cortical bone is thicker in the premolar regions compared to the molar regions, indicating potential differences in stability for implants placed in these areas. These findings emphasize the importance of understanding bone quality and thickness at different sites for implant success. As a result of this study, multiunit abutments exhibited superior fatigue performance compared to cemented abutments. There are several studies in the literature comparing the two abutment types [55,56,57]. In line with the results obtained in our study, multiunit abutments are considered advantageous over cemented abutments based on the available literature. While some studies have indicated that both multiunit and cement-retained restorations perform equally well in terms of technical complications [58], other research suggests that multiunit abutments offer benefits over cemented ones. Multiunit restorations provide predictable retrievability, unlike cemented restorations that may be susceptible to damage due to technical or biologic complications [59]. Additionally, multiunit crowns have demonstrated higher resistance to fracture following thermocycling and fatigue loading compared to cement-retained crowns [60]. Furthermore, multiunit abutments have shown superior results in terms of fluid and bacterial permeability compared to cemented abutments [55]. Peri-implant soft tissues have also exhibited more favorable responses to multiunit crowns when compared with cement-retained crowns [61]. Additionally multiunit prostheses offer advantages such as retrievability and avoidance of issues associated with excess cement over cement-retained prostheses [62].

FEA has emerged as a pivotal computational method in the field of dental research, particularly for investigating the biomechanical behavior of dental restorations, biomaterials, restorative techniques, and prosthetic designs. This numerical simulation technique allows researchers to model complex dental structures and predict their responses to various loading conditions, providing insights into stress distribution and mechanical performance under simulated functional scenarios. The primary advantage of FEA lies in its ability to dissect intricate geometries and material behaviors, enabling a detailed examination of stress patterns within dental biomechanics. By employing mathematical models, FEA can simulate how forces are transmitted through dental components, such as implants, crowns, and bridges, under different loading scenarios, including axial, lateral, and oblique forces. This capability is particularly valuable in understanding how these forces affect the longevity and stability of dental restorations. Typically, FEA operates under idealized conditions, which include assumptions such as the perfect alignment of components and isotropic, linear material properties. These assumptions simplify the complex interactions that occur in real-world scenarios, where factors such as misalignment, material anisotropy, and non-linear behavior can significantly influence the biomechanical responses of dental structures. For instance, the mechanical properties of dental materials can vary based on their composition, processing methods, and environmental conditions, which may not be fully captured in a standard FEA model. Moreover, the analysis presented in this study, which is based on just 1000 loading cycles, may not adequately reflect the dynamic and multifaceted nature of biomechanical interactions that occur in vivo. In clinical settings, dental restorations are subjected to a much higher number of loading cycles over their lifespan, influenced by factors such as mastication, bruxism, and varying occlusal forces. Consequently, limiting the analysis to a relatively low number of cycles may overlook critical aspects of material fatigue and failure mechanisms that could arise over time. To enhance the accuracy and relevance of future studies employing FEA, it is recommended that researchers validate their findings with in vivo experiments. Such validation would provide a more comprehensive understanding of how dental restorations perform under actual physiological conditions, accounting for the complexities of biological responses, patient-specific factors, and the dynamic nature of oral function. Additionally, increasing the number of loading cycles analyzed in FEA studies would allow for a more robust assessment of the long-term performance and durability of dental materials and designs. Moreover, the absence of diverse materials (only monolithic zirconia was used) in the study limits the understanding of how different materials might influence factors such as stress distribution, fatigue resistance, and overall implant success. The results of this study should be supported by studies covering a wider range of materials. Finally, it would be valuable to apply thermal analysis in future studies to understand how materials respond to thermal stress and to predict the long-term performance of dental restorations, especially in the context of masticatory forces that generate heat during function. Such studies would provide a more holistic understanding of how different factors influence the success of dental implants, ultimately leading to improved clinical outcomes and patient satisfaction.

## 5. Conclusions

The aim of this study is to address an important research gap. To this end, it aims to provide a comprehensive perspective on the complex rehabilitation of implant-supported multiunit and resin-cemented abutment restorations in cases where implants are placed at an angle, especially in cases where resorption is observed, and to provide valuable information to clinicians in clinical practice. Direct comparison of fatigue performance between resorbed and non-resorbed cases using different abutment types is limited in the literature. This study aimed to eliminate this gap. Thus, a contribution to the literature has been made.

The results obtained in the study are as follows:

- Implants with angulated placements demonstrated a reduction in fatigue performance, which is a critical consideration for clinical practice. Clinicians must be attentive to the angulation of implants, as excessive angulation may exacerbate mechanical complications and lead to reduced overall durability and function. Optimal implant placement, ideally in a more aligned orientation, is crucial to minimize adverse stress effects and enhance the long-term success of the implant.
- Multiunit abutments generally achieved superior fatigue performance compared to cemented abutment types, particularly in scenarios involving implant angulation. Clinicians must therefore pay close attention to the selection of abutment types, especially in cases where implants are not perfectly aligned. Multiunit abutments may provide a more robust solution in these situations by accommodating angulations and mitigating the adverse effects on fatigue performance. This can lead to improved long-term outcomes and reduce the risk of mechanical complications, ultimately contributing to the success and durability of the implant restoration.
- Molar regions exhibited lower fatigue performance compared to premolar regions due to their role as the primary zone for masticatory load. This reduced fatigue performance is primarily attributable to the significantly higher forces exerted during chewing in the molar area. Clinicians must carefully consider these factors when planning implant placements and restorations in the molar area. The elevated masticatory forces in this region necessitate the use of more robust and resilient implant components to withstand the increased stress and reduce the risk of mechanical failure.
- Finally, lower fatigue performance values were observed in cases of bone resorption, likely due to the reduced support from cortical bone surrounding the implants. Clinicians must pay close attention to the implications of bone resorption when planning and placing implants. In cases where significant bone loss is present, it is essential to evaluate the remaining bone structure carefully and consider potential bone augmentation procedures or the use of implants with designs specifically intended for compromised bone conditions.

## Figures and Tables

**Table 1:** Material specifications defined in the model.

Material	Young’s Modulus (E MPa)	Poisson’s Ratio (ν)	Shear Modulus (G Mpa)	References
Cortical bone	E_x_ 12,600 E_v_ 12,600 E_z_ 19,400	ν_xy_ 0.300 ν_yz_ 0.253 ν_xz_ 0.253 ν_yx_ 0.300 ν_zy_ 0.390 ν_zx_ 0.390	G_xy_ 4,850 G_yz_ 5.700 G_xz_ 5.700	[12]
Trabecular bone	E_x_ 1148 E_v_ 210 E_z_ 1148	ν_xy_ 0.055 ν_yz_ 0.010 ν_xz_ 0.322 ν_yx_ 0.010 ν_zy_ 0.055 ν_zx_ 0.322	G_xy_ 68 G_yz_ 68 G_xz_ 434	[12]
Mucosa	2.8	0.40		[13]
Titanium	110.000	0.33		[14]
Zirconia	210.000	0.30		[15]
Resin composite	12.000	0.33		[16]
Resin cement	5.100	0.27		[16]
Bone graft	3450	0.31		[17]

**Table 2:** Material values depending on Seegers method [20,21].

*σ’_f_*	*ε’_f_*	*b*	*c*
1554.71	0.35	−0.095	−0.69

## Data Availability

The raw data supporting the conclusions of this article will be made available by the authors on request.
